# Evaluation of 1-Year in-Home Monitoring Technology by Home-Dwelling Older Adults, Family Caregivers, and Nurses

**DOI:** 10.3389/fpubh.2020.518957

**Published:** 2020-10-02

**Authors:** Bruno Pais, Philipp Buluschek, Guillaume DuPasquier, Tobias Nef, Narayan Schütz, Hugo Saner, Daniel Gatica-Perez, Valérie Santschi

**Affiliations:** ^1^La Source, School of Nursing Sciences, HES-SO University of Applied Sciences and Arts Western Switzerland, Lausanne, Switzerland; ^2^DomoSafety SA, Lausanne, Switzerland; ^3^ARTORG Center for Biomedical Engineering Research, University of Bern, Bern, Switzerland; ^4^Department of Cardiology, University Hospital Bern, Bern, Switzerland; ^5^Idiap Research Institute, Martigny, Switzerland; ^6^School of Engineering, École Polytechnique Fédérale de Lausanne, Lausanne, Switzerland

**Keywords:** gerontechnology, patient satisfaction, monitoring technologies, home care, older people

## Abstract

**Introduction:** Population aging is increasing the needs and costs of healthcare. Both frailty and the chronic diseases affecting older people reduce their ability to live independently. However, most older people prefer to age in their own homes. New development of in-home monitoring can play a role in staying independent, active, and healthy for older people. This 12-month observational study aimed to evaluate a new in-home monitoring system among home-dwelling older adults (OA), their family caregivers (FC), and nurses for the support of home care.

**Methods:** The in-home monitoring system evaluated in this study continuously monitored OA's daily activities (e.g., mobility, sleep habits, fridge visits, door events) by ambient sensor system (DomoCare®) and health-related events by wearable sensors (Activity tracker, ECG). In the case of deviations in daily activities, alerts were transmitted to nurses via email. Using specific questionnaires, the opinions of 13 OA, 13 FC, and 20 nurses were collected at the end of 12-months follow-up focusing on user experience and the impact of in-home monitoring on home care services.

**Results:** The majority of OA, FC, and nurses considered that in-home sensors can help with staying at home, improving home care and quality of life, preventing domestic accidents, and reducing family stress. The opinion tended to be more frequently favorable toward ambient sensors (76%; 95% CI: 61–87%) than toward wearable sensors (Activity tracker: 65%; 95% CI: 50–79%); ECG: 60%; 95% CI: 45–75%). On average, OA (74%; 95% CI: 46–95%) and FC (70%; 95% CI: 39–91%) tended to be more enthusiastic than nurses (60%; 95% CI: 36–81%). Some barriers reported by nurses were a fear of weakening of the relationship with OA and lack of time.

**Discussion/Conclusion:** Overall, the opinions of OA, FC, and nurses were positively related to in-home sensors, with nurses being less enthusiastic about their use in clinical practice.

## Introduction

The population is aging in Europe and worldwide (1), including in Switzerland in which people over 65 years of age are anticipated to account for more than 25% of the population by 2050 (2). Population aging combined with the high costs of healthcare brings many challenges for healthcare systems, long-term care, and management of age-related chronic diseases (3). A recent study underlines that 79% of health costs in Switzerland are linked to chronic diseases (4, 5). With population aging, the prevalence of chronic diseases is increasing, resulting in rising healthcare needs and increasing costs. Additionally, both frailty and chronic diseases affecting older people reduce their ability to live independently. However, most older people prefer to age in their own homes (6, 7).

In response to these challenges, monitoring, and assistive technologies, such as emergency help systems, vital sign monitoring, or fall detection systems, can be a solution to support home care of older people to help them stay independent and active for a longer time (8–11). Currently, ambient sensors (12–15), also known as ambient living sensors, and wearable devices (16, 17) are used in the homes of older people to monitor changes in health status, to detect falls, or to monitor activities of daily living (18–23). Such technologies can allow older people to better connect and communicate with their healthcare professionals as well as with their families.

A literature review on monitoring technologies has suggested that a combination of monitoring technologies including ambient and wearable sensors technologies is probably the most effective solution in independently living older people (24). However, such an in-home monitoring system needs to be evaluated in a real-life setting to demonstrate the potential to prolong independent living of older people (24).

The objective of this 12-month observational study was therefore to evaluate the usability, functionality, and effects of a new in-home monitoring system—combining ambient and wearable sensors—among home-dwelling older adults (OA), their family caregivers (FC), and nurses for the support of home care, focusing on their end user experience and the impact of these technologies on the daily practice in home care service (25).

## Materials and Methods

### Study Design and Setting

This 12-month observational study was conducted among older people living independently at home and followed by nurses from NOMAD, the Neuchâtel public home care association, located in Switzerland, between January 2017 and July 2018. The study was approved by the Ethics Committee of the canton of Vaud, Switzerland (CER-VD ID: 2016-00762), and conducted based on principles declared in the Declaration of Helsinki. A written informed consent was obtained from all participating patients before study participation. We obtained the copyright holder permission to use and publish on the ambient sensor system (DomoCare®) by DomoSafety S.A.

### Study Participants and In-Home Monitoring System

Patients for participation were identified and recruited by NOMAD nurses in collaboration with a research assistant (BP) through the NOMAD database if they met the following inclusion criteria: (1) home-dwelling older adults (OA ≥70 years) living alone at home and without pets; (2) followed by nurses from NOMAD, Neuchâtel public home care association; (3) speak and read in French. Exclusion criteria were (1) severe cognitive impairment unable to follow study protocol (clock-drawing score ≥ 4); (2) skin problems, such as irritations, itching, serious redness; (3) undergoing dialysis; (4) not willing to comply with the study protocol; (5) unable to understand the study aim; (6) hospitalization planned in a short period of time.

After potential eligible patients' screening, patients who were likely to meet inclusion criteria were approached in person during a phone call or a visit of research assistant (BP), given an information letter if they expressed an interest in participating, and scheduled an appointment at home. Once the eligible patient agreed to participate and provided a written consent form, an in-home monitoring system comprising the ambient sensors [DomoCare®, DomoSafety S.A, Lausanne, Switzerland (26)] and wearable sensors (ECG, Activity tracker) was installed, respectively at home and on the patient's chest and wrist. In-home monitoring was conducted for 12 months.

### Data Collection and In-Home Monitoring System

During the 12 months of follow-up, the in-home monitoring system continuously monitored different OA's daily activities (e.g., mobility, sleep habits, fridge visits, door events) by ambient sensors (DomoCare®) and health-related events (e.g., physical activity and mobility, heart rate, skin temperature) by wearable sensors (Activity tracker, ECG). More precisely, the following data were recorded by wearable sensors: ECG signal, heart rate, heart rate variability, skin temperature, and respiration rate, as well as physical activity and mobility detected by accelerometer. The ECG sensor of type Preventice BodyGuardian was composed of a small and light battery powered device that was directly applied on the chest of the OA using a dry electrode and silicone-based adhesive patch. The data of the ECG sensor was automatically collected and transferred wirelessly from the sensor to a dedicated mobile phone and uploaded to servers for further analyses. The wearable Activity tracker, worn on the wrist of OA, was recorded and transmitted physical activity data (e.g., movement, number of steps) as well as heart rate.

Ambient motion sensors, installed in each apartment, recorded the OA's daily activities by passive infrared sensor (PIR) technology placed in the living room, bedroom, kitchen, and bathroom. Additional sensors were placed on the fridge and entrance door, measuring opening and closing of the door, and under the bed mattress, measuring bed presence, sleep cycles, respiration, and vital signs. The collected data were transmitted to servers for analysis by a base station. Data were then interpreted and subjected to trend analysis to detect changes in ADL and prevent changes in health status. In case of deviations in activities of daily living (e.g., changes in mobility), alerts were transmitted to nurses via email, using a report with weekly patient's activity graph and information summarizing the deviation detected by ambient sensors (e.g., decrease of patient's mobility for 3 days).

### Data Collection and Satisfaction of Home-Dwelling Older Adults, Family Caregivers, and Nurses

At the end of follow-up, specific self-administered mailed questionnaires were used to obtain users' satisfaction of OA, their family caregivers (FC), and nurses related to the in-home monitoring system and its impact on home care services. There were 20 nurses and 12 OAs. Nurses usually have care for several OAs and conversely OAs are usually cared for by more than one nurse. For each of the 20 nurses invited to respond to the questionnaire, an associated patient was randomly selected. Random selection was conducted by a statistician and restricted to combinations including the 12 OAs. This process had no impact on data collection.

Data were collected among 13 OA who completed the 1-year questionnaire, 13 family caregivers who play a central role as full partner in care and well-being of OA, and 20 nurses. Semi-structured interviews face-to-face or phone calls were conducted by a research assistant (BP) to complete the answers of the questionnaires described below.

The questionnaires filled by OA and nurses were based on the French version of the instrument titled the Quebec User Evaluation of Satisfaction with assistive Technology (QUEST) by Demers et al. (27), and the questionnaire filled by FC was based on the caregiver quality of life scale developed and validated in France from data of the Pixel studies (28). These three questionnaires included open- and closed-ended response options, with additional questions on OA, FC, and nurse opinions', satisfaction, and practical experiences related to the in-home monitoring.

The questionnaires assessed 4 items: (1) opinion on the usefulness of ambient and wearable sensors; (2) satisfaction of OA, FC, and nurse with ambient and wearable sensors; (3) impact of sensors on the relationship between OA, FC, and nurse; (4) impact on in home care practice (integration and barriers). Answers to questionnaires were dichotomous (yes/no) or graduated on a five-point Likert scale, ranging from “a lot” to “not at all.”

The construction, relevance, and comprehensiveness of questionnaires were assessed among five OA and one NOMAD nurse to ensure that they were easily understandable, well-defined, and accurately addressed the goals of the study.

### Statistical and Data Analysis

Descriptive statistics were used to present baseline characteristics of OA and results related to ambient and wearable sensors as number, percentage, and score average. Overall opinion was calculated as weighted percentage. For the main results, 95% binomial confidence intervals (CI) were built around this percentage (command stata: cii proportion), i.e., the overall opinion and satisfaction of participants. All statistical analyses were performed using Stata software version 15.0 (Stata Corp, College Station, TX, USA) and Excel.

## Results

**Figure 1:**
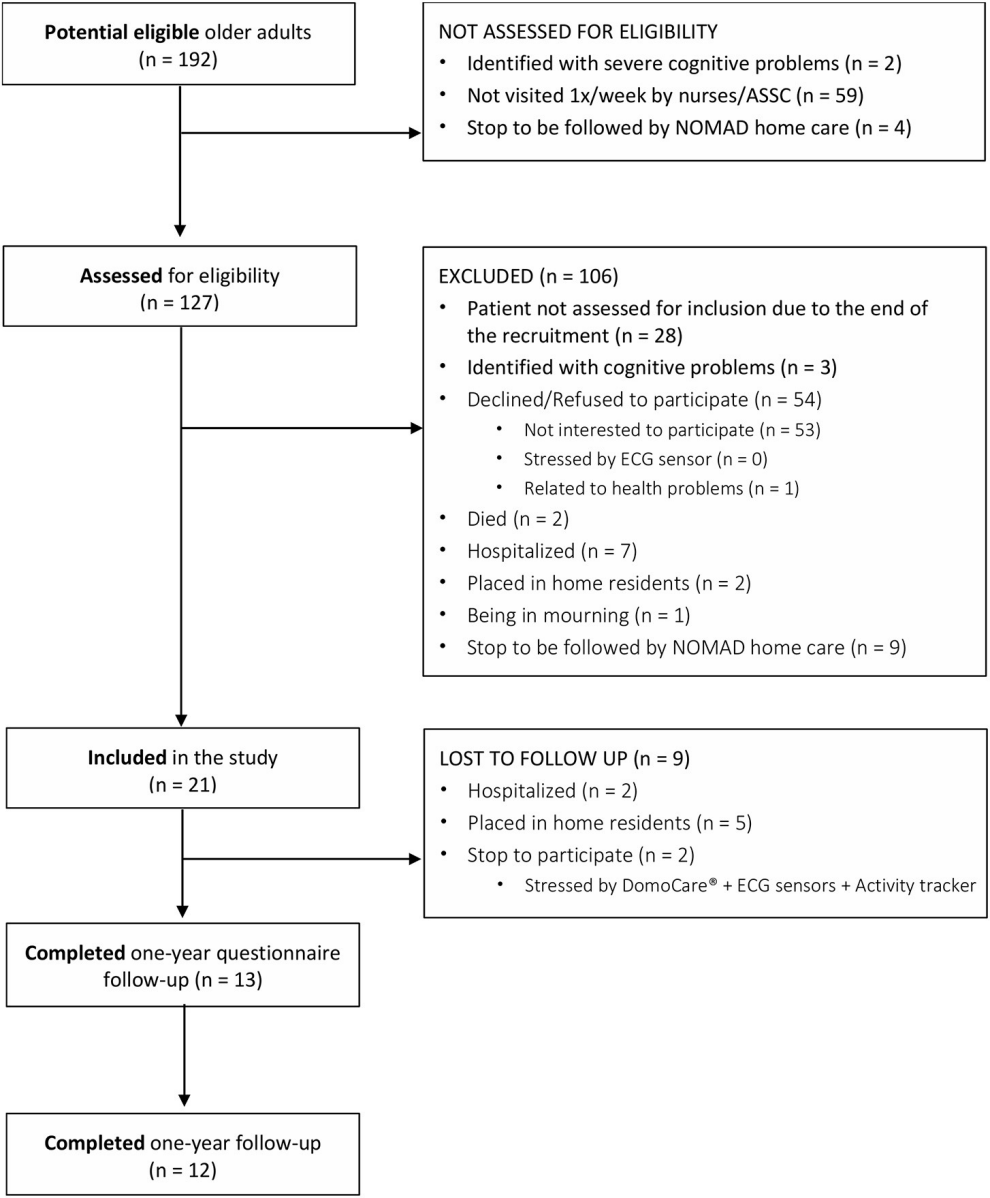
Flow diagram of home-dwelling older adults.

The study design and flow-chart of patients are presented in Figure 1. Among the 192 potential eligible OA, 127 OAs were assessed for eligibility, 54 (42.5%) refused to participate, and 52 (40.9%) were excluded during the process (e.g., hospitalization, cognitive problems, death, placed in home residents). A total of 21 were included in the study, and 12 completed the 1-year of follow-up. The remaining 9 patients could not be contacted as a result of hospitalization and institutionalization. A total of 13 patients completed the 1-year questionnaire follow-up.

### Characteristics of Patients

The baseline characteristics of the 21 included patients are shown in Table 1. The mean age was 85 years, half were men, and half received home care for more than 1 year. Patients took on average 9 drugs daily, 90% of patients were treated with 5 drugs or more per day, and 81% reported using a weekly pillbox to facilitate their drug intake. During the follow-up, 43% of patients were hospitalized at least once. Most patients were diagnosed with comorbidities, such as cardiovascular diseases, hypertension, dyslipidaemia, and diabetes.

**Table 1:** Baseline characteristics of included older adults.

**Characteristics**	
**Number of patients**	**21**
Men/women, *n*	11/10
Mean age, years (*SD*) [range]	85 (7) [72–96]
Mean body mass index, kg/m^2^ (*SD*)	26 (5)
Marital status	
Single, *n* (%)	1 (5%)
Married, *n* (%)	0 (0%)
Divorced, *n* (%)	2 (10%)
Widowed, *n* (%)	18 (86%)
Nationality	
Swiss, *n* (%)	19 (90%)
No-Swiss, *n* (%)	2 (10%)
**Comorbidities**	
Current smoker (≥1 cigarette/day), *n* (%)	0 (0%)
Cardiovascular diseases, *n* (%)	16 (76%)
Diabetes mellitus, *n* (%)	4 (19%)
Hypertension, *n* (%)	15 (71%)
Dyslipidaemia, *n* (%)	8 (38%)
Chronic kidney disease, *n* (%)	0 (0%)
**Number of drugs**, ***n*** **(SD) [range] [median]**	**9 (5) [3–20] [9]**
Polymedication (5 drugs or more), *n* (%)	19 (90%)
Using a weekly pillbox, *n* (%)	17 (81%)

*SD, standard deviation*.

### Overall Opinion and Satisfaction

The majority of OA, FC, and nurses considered that in-home sensors (ambient and wearable) can help staying at home, improving home care and quality of life, preventing domestic accidents, and reducing family stress (Table 2). The opinion tended to be more frequently favorable toward ambient sensors (76%; 95% CI: 61–87%) than toward Activity tracker (65%; 95% CI: 50–79%) and ECG (60%; 95% CI: 45–75%). On average, OA (74%; 95% CI: 46–95%) and FC (70%; 95% CI: 39–91%) tended to be more enthusiastic than nurses (60%; 95% CI: 36–81%).

**Table 2:** Opinion on the usefulness of ambient (DomoCare®) and wearable sensors (Activity tracker, ECG) to help staying at home, improving home care, improving quality of life, preventing domestic accidents, or reducing family stress.

	**Older adults (*n* = 13) (%)**	**Family caregivers (*n* = 13) (%)**	**Nurses (*n* = 20) (%)**	**Average (*n* = 46) (%)**
DomoCare®	82	80	69	76
Activity tracker	63	69	63	65
ECG sensor	76	60	49	60
Average	74	70	60	

### Opinion and Satisfaction on the Ambient Sensor System (DomoCare®)

**Figure 2:**
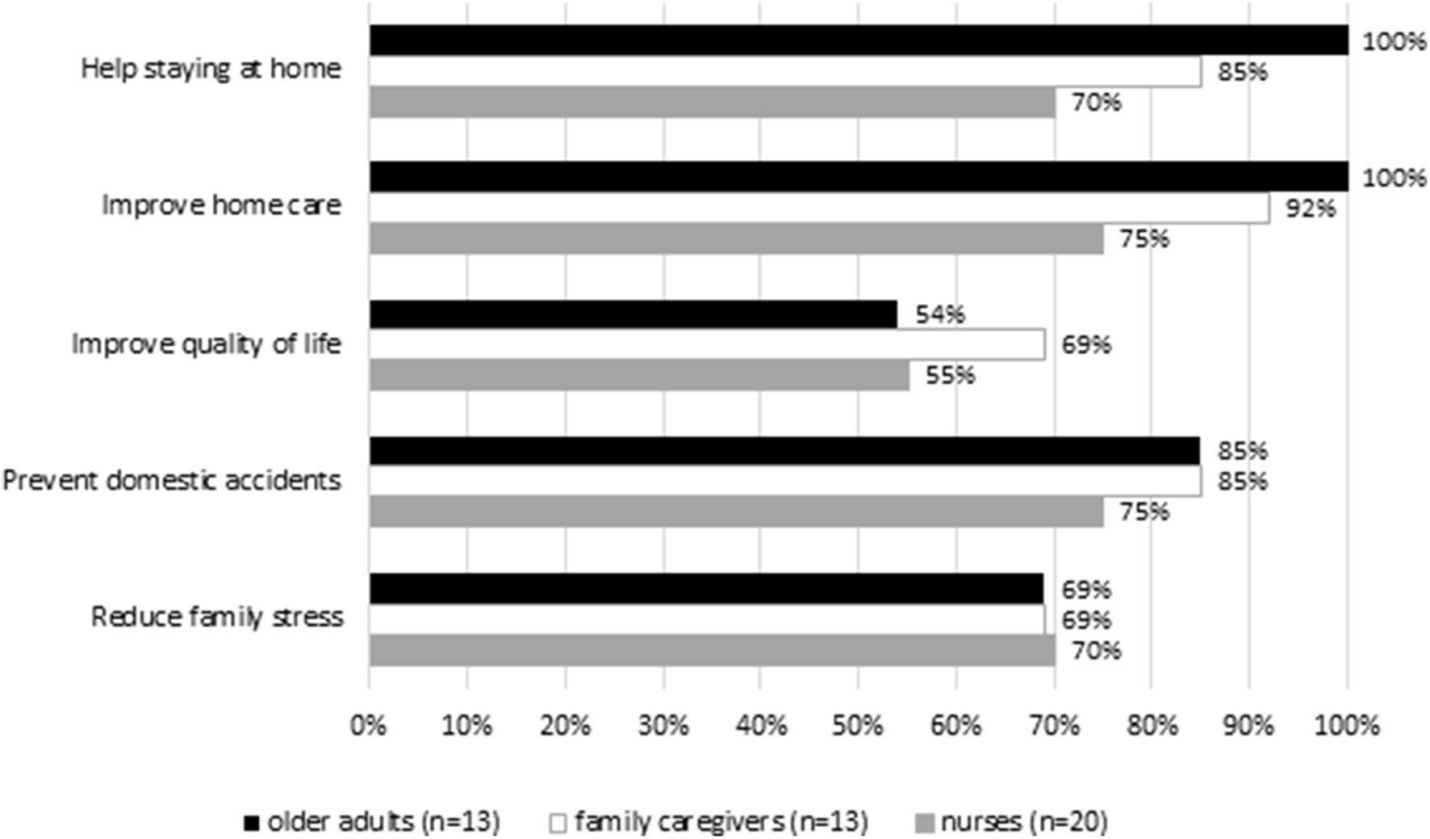
Opinion on the ambient sensor system (DomoCare®).

As shown in Figure 2, most of OA and FC rated higher ambient sensors (DomoCare®) in helping staying at home (OA: 100%, FC: 85%), improving home care (OA: 100%; FC: 92%), preventing domestic accidents (OA: 85%; FC: 85%), and reducing family stress. The FC (69%) rated higher in-home sensors in improving quality of life compared to OA (54%) and nurses. More than half of OA (69%), FC (69%), and nurses (70%) considered ambient sensors can help reducing family stress. Overall, the majority of OA were satisfied with DomoCare®. Nurses tended to be less enthusiastic than OA, particularly regarding the technical (e.g., dimension, solidity) and practical settings of ambient sensors (e.g., ease of installation and use; Supplementary Figure 1).

### Opinion and Satisfaction on the Wearable Sensors (ECG and Activity Tracker)

**Figure 3:**
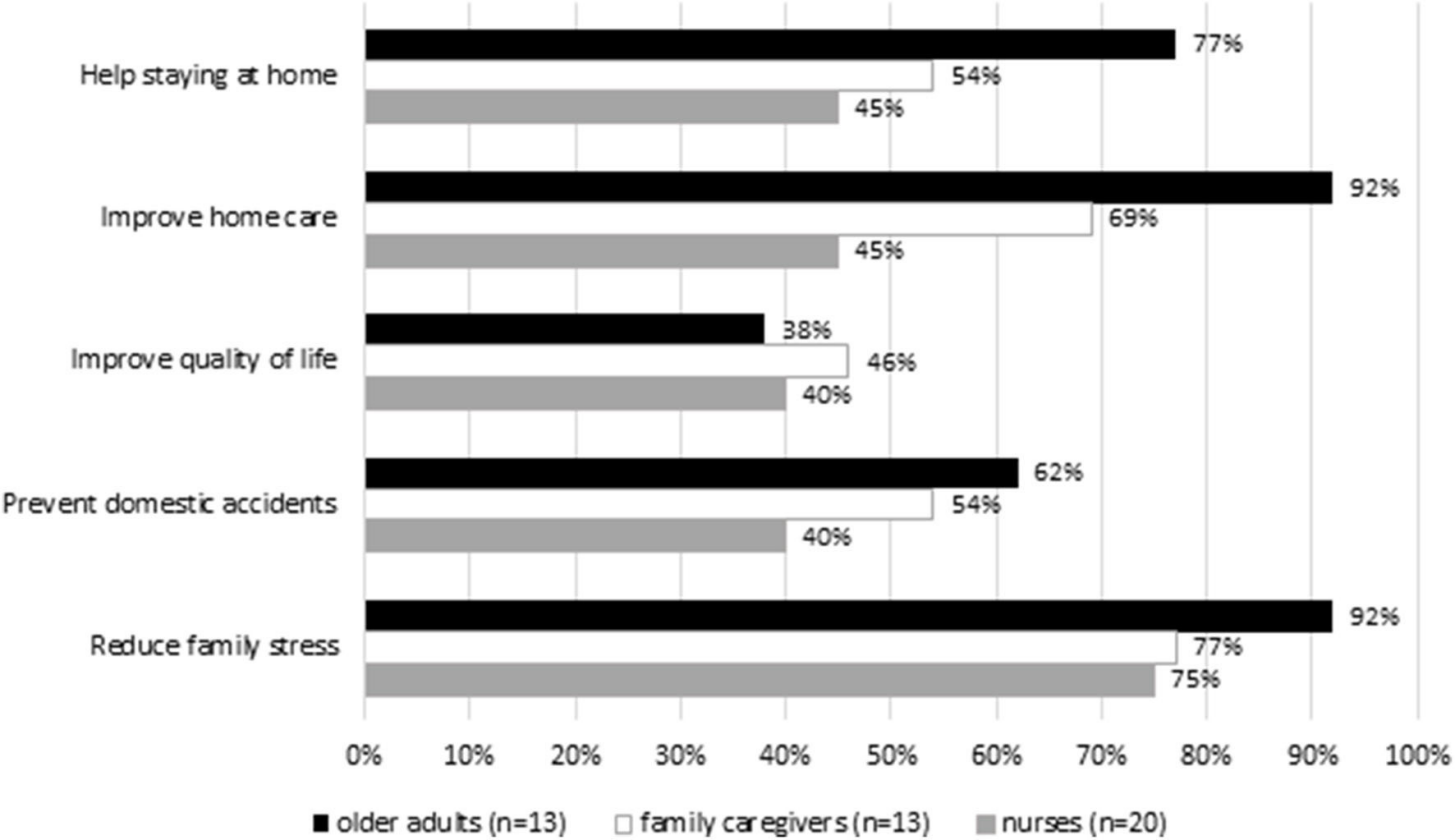
Opinion on the wearable sensor (ECG).

**Figure 4:**
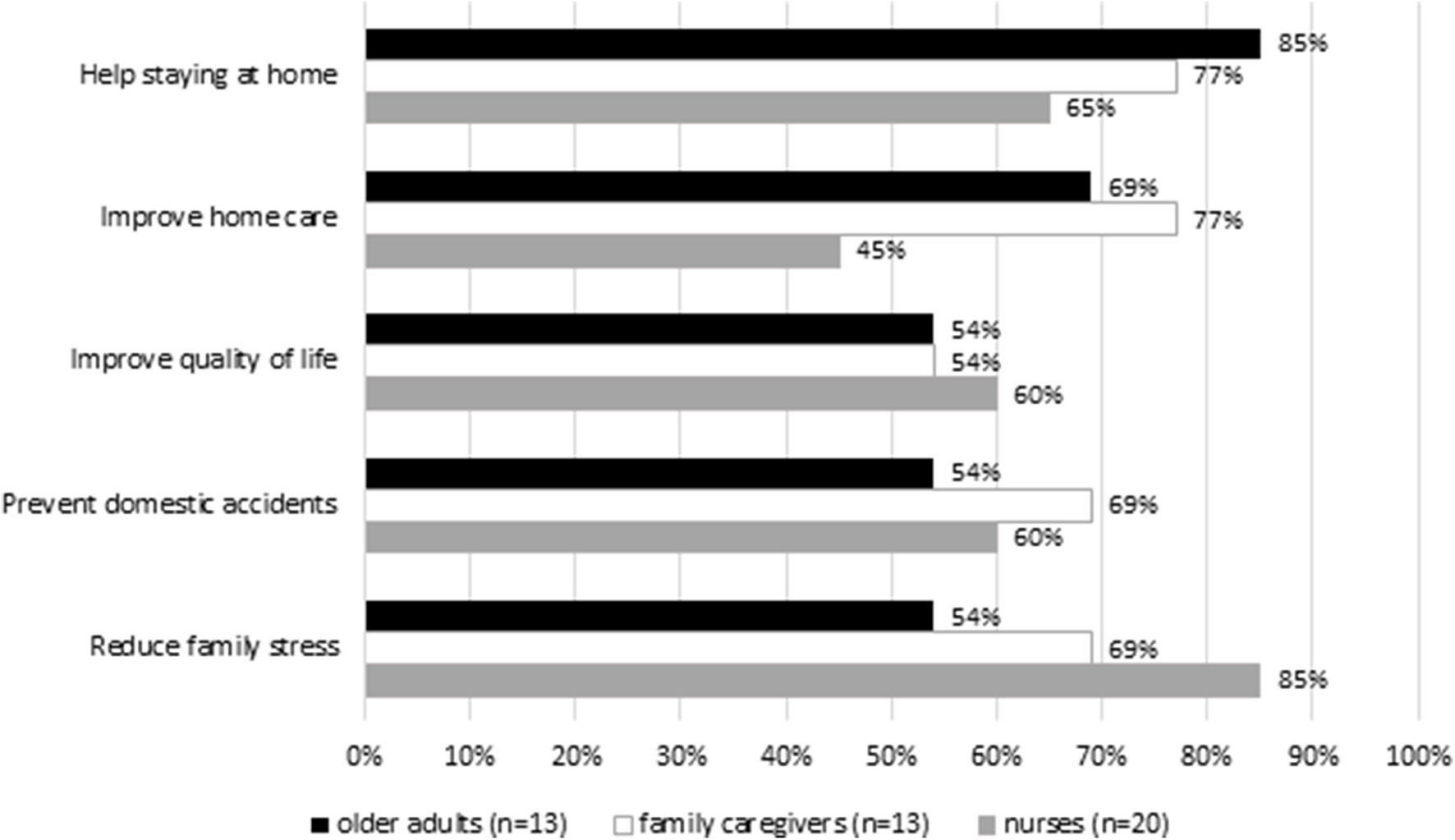
Opinion on the wearable sensor (Activity tracker).

Regarding the wearable sensors (Figures 3, 4), most of OA and FC rated higher activity tracker in helping staying at home (OA: 85%; FC: 77%) and improving home care (OA: 69%; FC: 77%) compared to ECG. Most nurses rated higher activity tracker in improving quality of life (60%) and reducing family stress (85%). Regarding the ECG, the majority of the OA (92%), FC (77%), and nurses (75%) underlined that ECG can help reduce family stress. More than half of OA, FC, and nurses were skeptical regarding the prevention of domestic accidents for the wearable sensors [activity tracker (OA: 54%; FC: 69%; nurses: 60%); ECG (OA: 62%; FC: 54%; nurses: 40%)]. Overall, OA and nurses were satisfied with the Activity tracker and ECG. However, nurses tended to be less enthusiastic than OA, particularly with ECG, reporting that wearable sensors should be made smaller, lighter, and more comfortable (Supplementary Figures 2, 3).

### Events and Nurse Interventions

A total of 76 events were detected by DomoCare® (on average 4 events/patients; range 0–12 events). Each of them generated a preventive report validated by a research assistant (BP) and was transferred to NOMAD nurses by email. Events corresponded to changes in mobility behavior (26/76, 34%), in toilet usage frequency (24/76, 32%), in fridge usage (11/76, 14%), in entrance door usage (4/76, 5%), in time spent out of home (2/76, 3%), and in time spent in bed (9/76, 12%). For all events, nurses followed up with a home visit.

### Integration of In-Home Monitoring Technology in Home Care Practice

Overall, the majority of nurses considered in-home sensors (DomoCare®: 65%; ECG: 55%; Activity tracker: 70%) easily implementable in their practice. However, nurses considered work overload, lack of time, and cumbersome procedures, especially for DomoCare® and ECG, and fear of weakening of the relationship with OA, as barriers to implementing in-home sensors (Supplementary Figure 4). Less than 50% of nurses reported lack of remuneration as a barrier. Finally, the majority of OA, FC, and nurses reported that they would like to continue using in-home sensors in case of insurance reimbursement.

## Discussion

This 12-month observational study showed that the majority of OA, FC, and nurses valued in-home monitoring systems including ambient and wearable sensors, notably to help staying at home, improving home care, preventing domestic accidents, and reducing family stress. On average, OA and FC tended to be more enthusiastic than nurses about this in-home monitoring system. Moreover, some barriers were reported by nurses, such as a fear of weakening of the relationship with OA and an excessive surveillance. Overall, the opinions of OA, FC, and nurses were positively related to in-home technology, with nurses being less enthusiastic about their use in clinical practice.

### Comparison With Other Studies

Our findings suggested a variety of benefits and positive potential impacts of in-home monitoring on quality of life of OA and nursing home care services. As described by previous studies (29–34), the majority of OA, FC, and nurses were not familiar with in-home monitoring technologies. However, they considered that such technologies can be relevant in the future by improving the quality of care among OA living independently at home. Moreover, the previous studies underlined that the acceptance and the use of in-home technologies by OA were often influenced by social network and pointed work overload of nurses, cost, and lack of funding to be barriers to implement in-home monitoring technologies.

In our study, OA and FC tended to be more enthusiastic than nurses regarding new in-home technologies. These results are consistent with other studies (29–34) showing that OA perceived in-home technologies as a viable home care solution, which can prolong their time living at home, whereas nurses had concerns that such technology could weaken their relationship with OA and worsen their work conditions.

### Strengths and Limitations of the Study

Strengths of our study are mainly (1) the close collaboration between OA, FC, nurses, researchers, and engineers; (2) the innovative use of assistive and digital technologies designed to support independent community older adults; and (3) the collection data on the use of in-home monitoring devices. Furthermore, all OA who completed the 12-month follow-up, all nurses, and all FC filled out the questionnaires. However, we acknowledge some limitations to our study. Due to logistical and financial reasons, the study sample was small. There was also a high loss to follow-up notably due to hospitalization and moving to a nursing home, which are frequent events among this type of patients. Furthermore, for logistical reasons, the study was conducted in only one place, which limits its external validity. Further studies are therefore needed to evaluate the transferability of our findings to other regions and populations. We did not use a specific theoretical acceptance model in this study, but further studies would gain from doing so. Finally, we tested one in-home monitoring system and a given set of sensors, and our findings may therefore not apply to other systems or individual sensors.

### Future Perspective

In conclusion, OA, FC, and nurses were very or quite positively related to DomoCare®, Activity tracker, and ECG sensors and reported that in-home monitoring technologies may facilitate home care and opened good perspectives for use in home care practice. Further studies, at a larger scale, are needed to evaluate how this type of in-home monitoring can help patients stay longer at home, improve health care management, and reduce healthcare costs. Further, some manufacturing improvements (e.g., development of sensors that are smaller, lighter, and more user-friendly and comfortable for OA, as well as advances in machine learning for detection of specific events at home) and training of nurses in the use of these monitoring systems should be considered, to ease their use, increase comfort of end-users, and preserve and strengthen the relationship between OA and nurses are key for implementing these new technologies in nursing home care practice (32–39).

## Data Availability Statement

All datasets generated for this study are included in the article/Supplementary Material.

## Ethics Statement

The study was reviewed and approved by CER-VD: Cantonal Ethics Committee of Vaud on Research involving humans. The patients/participants provided their written informed consent to participate in this study.

## Author Contributions

BP, PB, GD, TN, NS, HS, DG-P and VS designed and planned the study. DomoSafety S.A. installed and maintained the wearable sensors (ECG, Activity tracker) and ambient sensor system (DomoCare®) monitoring the participants. BP and NS measured participants. BP and VS analyzed data from the questionnaires filled out by the participants, their families and nurses at the end of follow-up, and wrote the manuscript. All authors reviewed and approved the final manuscript.

## Conflict of Interest

PB and GD are employed by DomoSafety S.A., which is the manufacturer of the displayed ambient sensor system (DomoCare^®^). The remaining authors declare that the research was conducted in the absence of any commercial or financial relationships that could be construed as a potential conflict of interest.
