# Supplementary material for: Evaluation of 1-Year in-Home Monitoring Technology by Home-Dwelling Older Adults, Family Caregivers, and Nurses
Source: Front Public Health. 2020 Oct 2;8:518957. doi: 10.3389/fpubh.2020.518957 (PMC7562920; doi:10.3389/fpubh.2020.518957)
Supplement: Supplementary file 1 [file Data_Sheet_1.DOCX]

Supplementary Material

# SUPPLEMENTARY FIGURE LEGENDS

# Supplementary Figure 1: Satisfaction related to ambient sensor system (DomoCare^®^)

# Supplementary Figure 2: Satisfaction related to wearable sensor (ECG)

# Supplementary Figure 3: Satisfaction related to wearable sensor (Activity tracker)

# Supplementary Figure 4: Barriers to integration of ambient and wearable sensors

# Supplementary Figure 1: Satisfaction related to ambient sensor system (DomoCare^®^)

**Supplementary Figure 2:** Satisfaction related to wearable sensor (ECG)

**Supplementary Figure** **3:** Satisfaction related to wearable sensor (Activity tracker)

# Supplementary Figure 4: Barriers to integration of ambient and wearable sensors
