# Supplementary figures and images for: Evaluation of 1-Year in-Home Monitoring Technology by Home-Dwelling Older Adults, Family Caregivers, and Nurses
Source: Front Public Health. 2020 Oct 2;8:518957. doi: 10.3389/fpubh.2020.518957 (PMC7562920; doi:10.3389/fpubh.2020.518957)

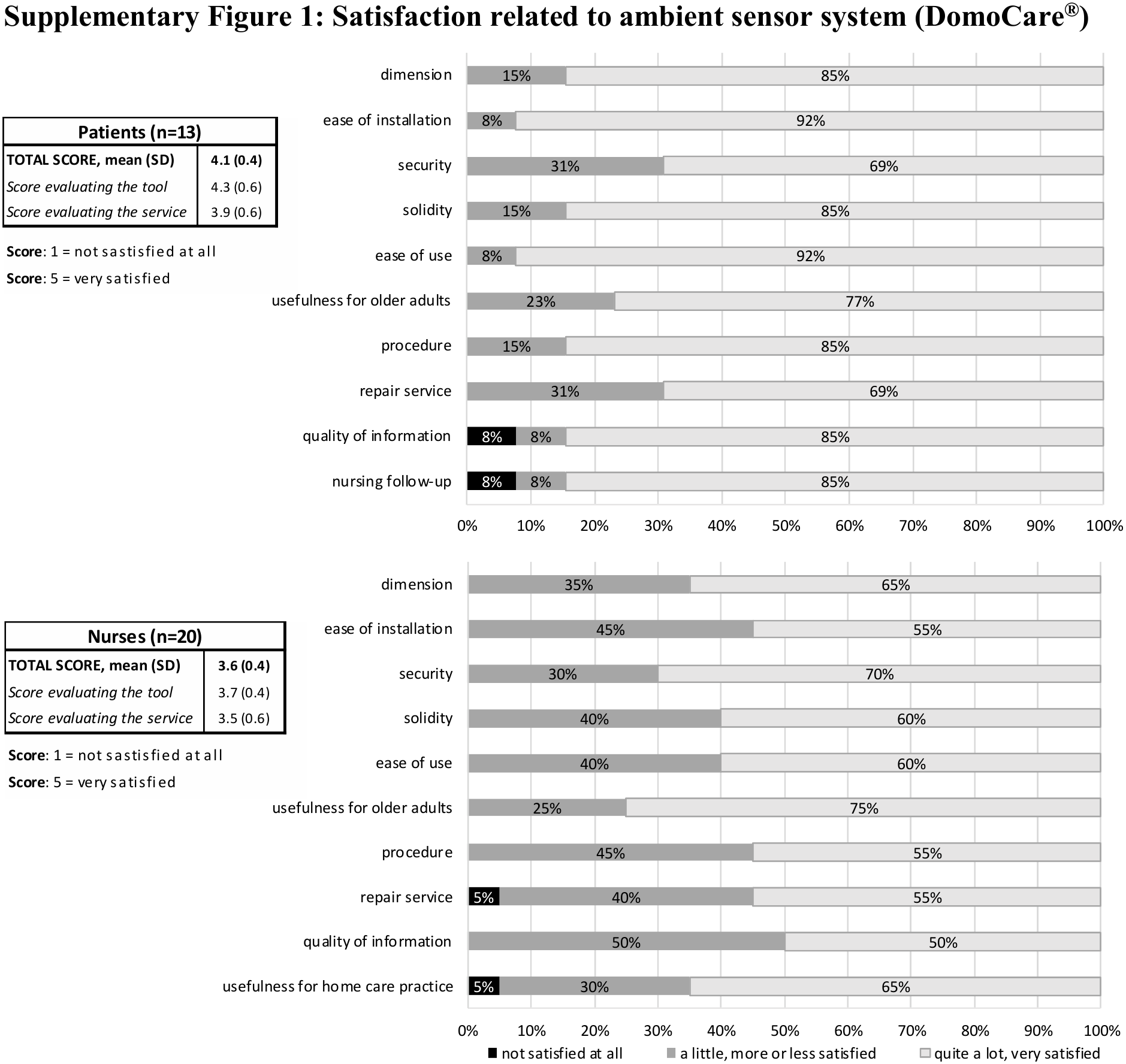

Supplement: Supplementary file 2 [file Image_1.TIF]

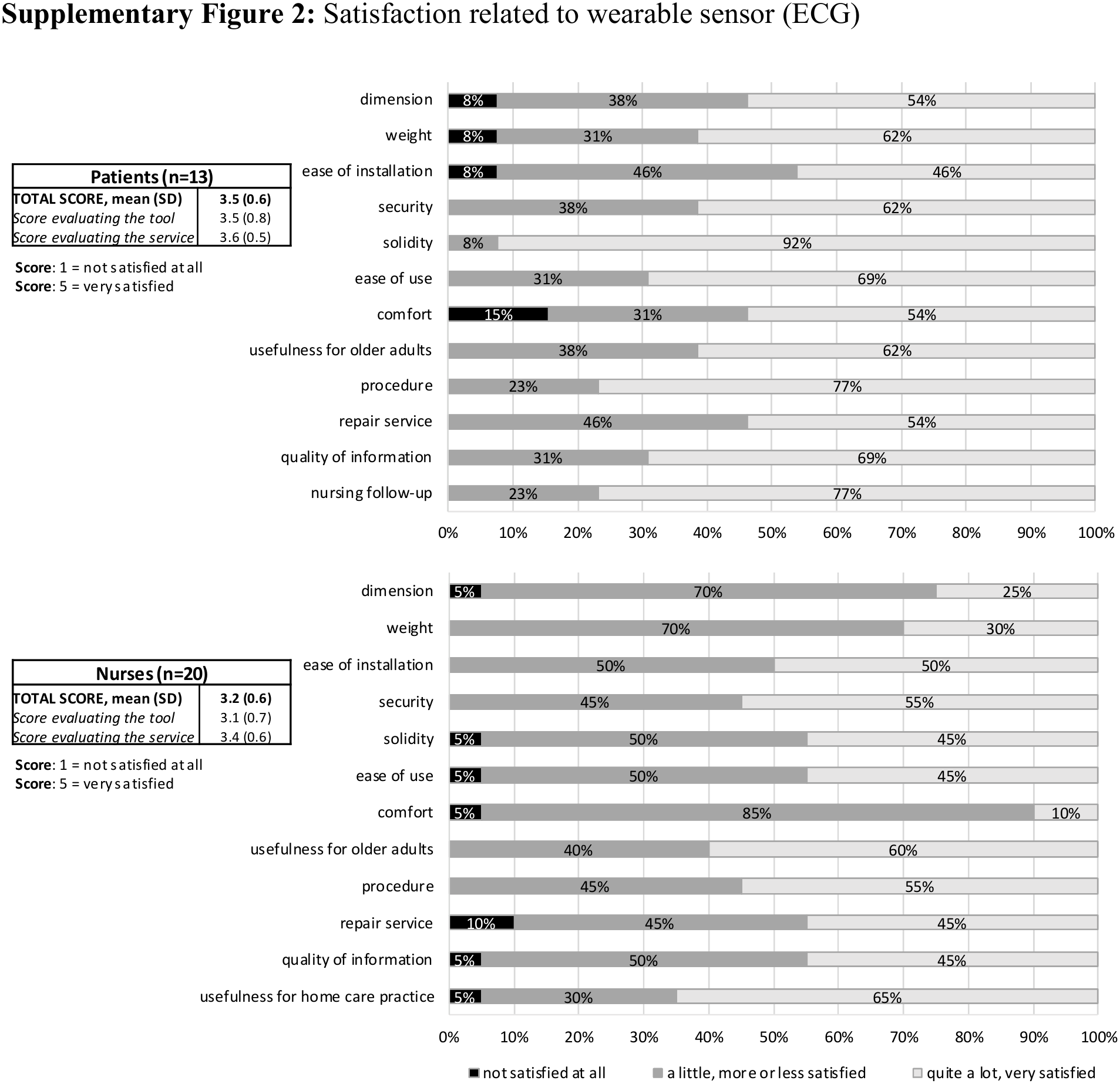

Supplement: Supplementary file 3 [file Image_2.TIF]

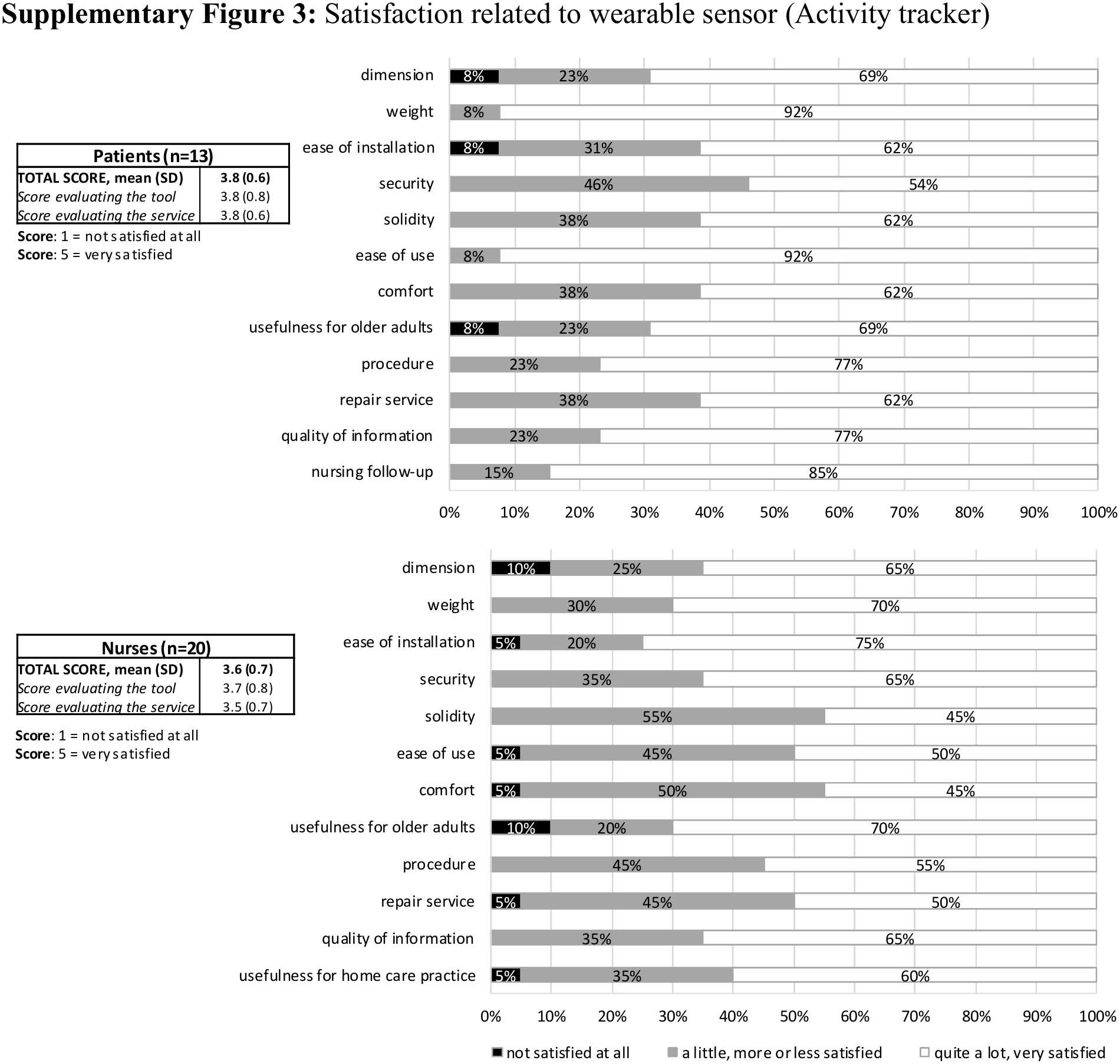

Supplement: Supplementary file 4 [file Image_3.TIF]

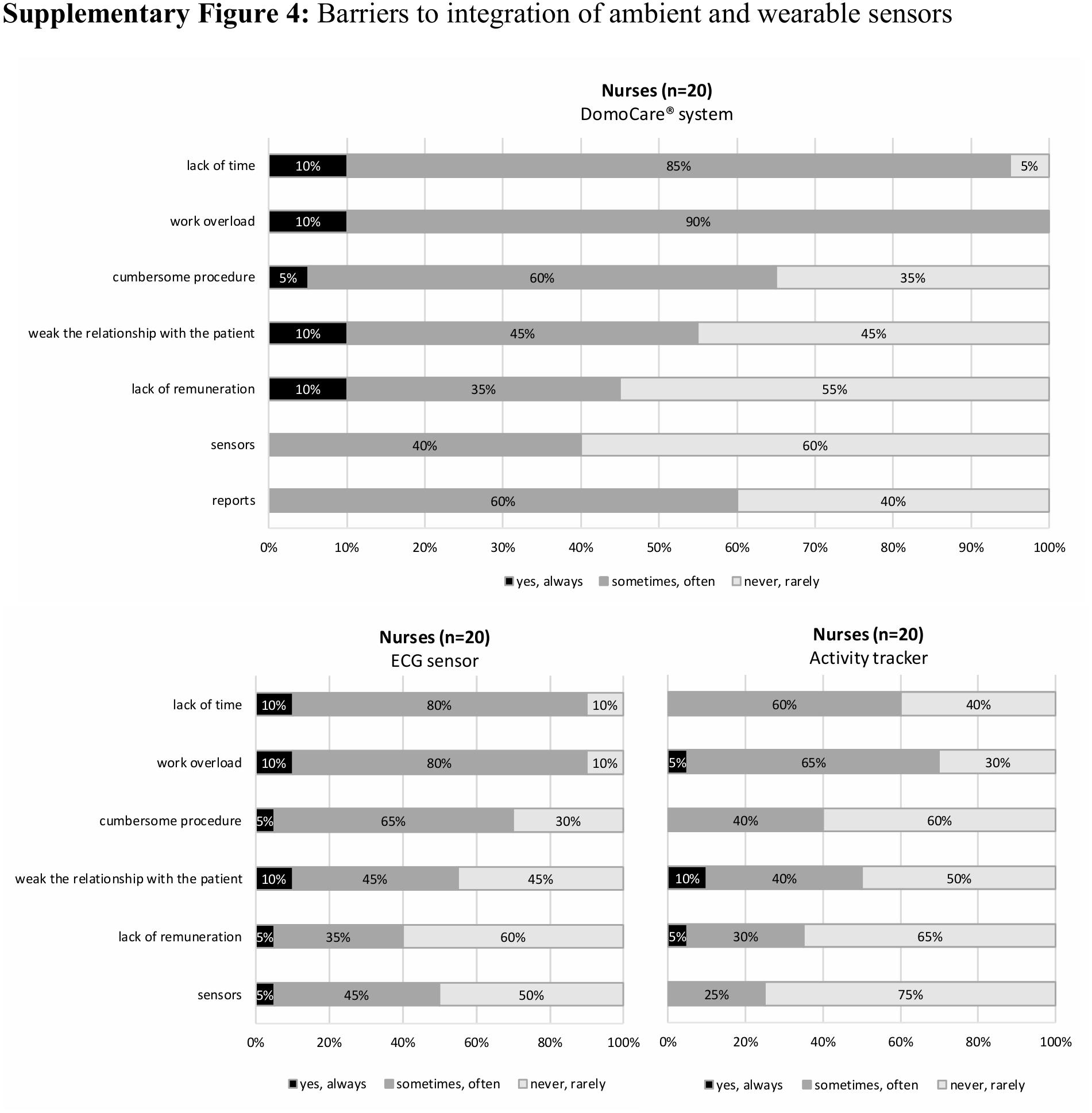

Supplement: Supplementary file 5 [file Image_4.TIF]
